# Electroporation as a Solvent-Free Green Technique for Non-Destructive Extraction of Proteins and Lipids From *Chlorella vulgaris*

**DOI:** 10.3389/fbioe.2020.00443

**Published:** 2020-05-13

**Authors:** Tina Eleršek, Karel Flisar, Blaž Likozar, Marina Klemenčič, Janvit Golob, Tadej Kotnik, Damijan Miklavčič

**Affiliations:** ^1^Department of Genetic Toxicology and Cancer Biology, National Institute of Biology, Ljubljana, Slovenia; ^2^Faculty of Electrical Engineering, University of Ljubljana, Ljubljana, Slovenia; ^3^National Institute of Chemistry, Ljubljana, Slovenia; ^4^Faculty of Chemistry and Chemical Technology, University of Ljubljana, Ljubljana, Slovenia

**Keywords:** electroporation, lipid extraction method, microalgae, protein extraction and processing, solvent-free extraction

## Abstract

Proteins extracted from microalgae for food, personal care products and cosmetics must be of high purity, requiring solvent-free extraction techniques despite their generally considerably lower protein yield and higher energy consumption. Here, three such approaches for green extraction of proteins from *Chlorella vulgaris* were evaluated: ultrasound, freeze-thawing, and electroporation; chemical lysis was used as positive control (maximal achievable extraction), and no extraction treatment as negative control. Compared to chemical lysis, electroporation yielded the highest fraction of extracted protein mass in the supernatant (≤27%), ultrasound ≤24%, and freeze-thawing ≤15%. After a growth lag of several days, electroporated groups of algal cells started to exhibit growth dynamics similar to the negative control group, while no growth regeneration was detected in groups exposed to ultrasound, freeze-thawing, or chemical lysis. For electroporation as the most efficient and the only non-destructive among the considered solvent-free protein extraction techniques, simultaneous extraction of intracellular algal lipids into supernatant was then investigated by HPLC, proving relatively low-yield (≤7% of the total algal lipid mass), yet feasible for glycerides (tri-, di-, and mono-) as well as other fatty acid derivatives. Our results show that electroporation, though lower in extraction yields than chemical lysis or mechanical disintegration, is in contrast to them a technique for largely debris-free extraction of proteins from microalgae, with no need for prior concentration or drying, with feasible growth regeneration, and with potential for simultaneous extraction of intracellular algal lipids into the supernatant.

## Introduction

Green, solvent-free, and preferably non-destructive extraction of natural chemical compounds from microorganisms is among the key concepts in meeting the 21st century challenges of protecting both the environment and the consumers. Green extraction is the umbrella term for innovative energy-efficient and environment-friendly extraction techniques that minimize the use of conventional solvents, replacing them with biosolvents and/or renewable natural products, and thus enabling production of safe and high-quality extracts (Chemat et al., 2012). If the extraction process is designed as to avoid the use of solvents altogether, this renders both the extracts and the leftovers free of solvent residuals, reducing both the health risk to consumers and the environmental footprint, as well as raising the extract purity to the levels required for food, personal care and cosmetics. Finally, if the extraction method is refined to also be non-destructive, so that the population of microorganisms from which the compounds are extracted retains the ability for cell growth regeneration, this reduces the amount of waste resulting from the extraction and thus further benefits the environmental footprint.

Naturally occurring algae are of great importance for aquatic ecosystems stability, O_2_ emission and CO_2_ sequestration, and are already established in wastewater treatment and in some biotechnological applications (Becker, 2007; Aschemann-Witzel et al., 2013). It is gradually also becoming evident that microalgae are promising organisms for sustainable biotechnological processes in which bio-refinery concepts are integrated with natural (sun, water, CO_2_), renewable (algal biomass), and/or recyclable (wastewater, nutrients) components (Golberg et al., 2016; Buchmann et al., 2019). In particular, microalgae can serve as a potential source of energy (Hannon et al., 2010), food (Draaisma et al., 2013), feed (Skrede et al., 2011), cosmetics, and pharmaceuticals (Olaizola, 2003a; Olaizola, 2003b) due to their higher photosynthetic efficiency, higher biomass production and faster growth compared to other energy crops (Widjaja et al., 2009). Microalgae can also be converted directly into energents such as biodiesel, which makes them a promising source of renewable energy (Gouveia and Oliveira, 2009; Halim et al., 2012). Moreover, microalgae are an attractive food/feed and food-supplement source, as they are rich in proteins, peptides, carbohydrates, lipids, omega 3 fatty acids, trace elements and other essential nutrients with protective and detoxifying roles (vitamins, minerals, pigments) (Wijffels et al., 2010; Gong and Jiang, 2011; Cuellar-Bermudez et al., 2015).

Under certain conditions, protein content in microalgae can represent as much as 60% of the biomass (Becker, 2007; Draaisma et al., 2013). Cells that produce these high-value proteins can be grown under controlled environmental conditions in photobioreactors (Walker et al., 2005) without adverse effects to the environment (Pulz and Gross, 2004). Microalgal proteins are of high quality and comparable to conventional plant proteins (FAO/WHO, 1973; Becker, 2007). In comparison to conventional protein-rich crops, microalgae have higher areal productivity, they can be grown in sea water and thus do not require arable land, exhibit no need for pesticides, are generally easily cultivated, and have no adverse effect on biodiversity (Godfray et al., 2010; Tilman et al., 2011).

Microalgae as protein source for food, however, have certain drawbacks. Similarly to plant crops, all algal products are poorly digested by humans (and all other non-ruminant animals), because algae (as plants) possess a rigid cell wall composed predominantly of cellulose. For effective availability of algal proteins in food, a post-harvesting treatment resulting in disruption of the cell wall must therefore be employed (Safi et al., 2012). For microalgae to become an established and widely used protein source in nutrition, these post-harvesting treatments must be acceptable economically (McMillan et al., 2013) and from the aspect of the resulting food microstructure, which affects the bioavailability of nutrients including proteins (Parada and Aguilera, 2007). But most importantly, the proteins extracted from microalgae for food – as well as for personal care products and cosmetics – must be of high purity, requiring solvent-free extraction techniques despite their generally lower protein yield and higher energy consumption. This clearly disqualifies chemical extraction techniques.

Among physical extraction techniques, bead-milling, high-pressure homogenization, ultrasonication, and freeze-thawing have all proved effective in protein extraction from microalgae (Hopkins, 1991; Middelberg, 1995; Doucha and Livansky, 2008; Günerken et al., 2015; Postma et al., 2015), and ultrasonication was also found effective in lipid extraction (Prabakaran and Ravindran, 2011). However, all these approaches generally result in cell lysis and disintegration. As a consequence, they all yield a mix of extracted proteins and structural debris of the microalgae (lipids from their cellular and intracellular membranes and the fragments of their cell wall), affecting the solubilization of the extracted proteins (Shen et al., 2008), as well as necessitating further fractionation and purification (Vanthoor-Koopmans et al., 2013).

In contrast, in electroporation, where exposure of cells to short high-voltage electric pulses is used to increase their permeability (Kotnik et al., 2019), pulse amplitude and duration highly optimizable for the permeabilizing effect to be limited and reversible, with the cells retaining their viability and integrity, so that the extract is largely free of the structural debris (Kotnik et al., 2015). Further fine*-*tuning of pulse parameters can also increase the selectivity of extraction to proteins or lipids (Coustets et al., 2013; Grimi et al., 2014); the reports of electroporation-based extraction of various biomolecules from various microalgae are summarized in Table 1. In comparison to ultrasonication and freeze-thawing, as well as high-pressure homogenization, electroporation was also reported to be the most economical in energy consumption (Wijffels et al., 2010; Goettel et al., 2013). And finally, when reversible and thus non-destructive, electroporation allows for the microorganisms to regenerate after extraction, reducing the amount of waste and thus the burden on the environment.

**TABLE 1:** Reports of electroporation-based extraction techniques from microalgae.

**References**	**Species**	**Extracted molecules**	**Voltage amplitude (kV)**	**Distance between electrodes (mm)**	**Pulse duration (ms)**	**Number of pulses**	**Pulse repetition frequency (Hz)**
Coustets et al., 2013	*C. vulgaris, N. salina*	Proteins	1.8	3 or 6	2	15	NR
Grimi et al., 2014	*Nannochloropsis* sp.	Proteins	20	NR	1–4	NR	NR
Coustets et al., 2015	*C. vulgaris, H. pluvialis, N. salina*	Proteins	1.8	3 or 6	2	9	NR
Postma et al., 2016	*C. vulgaris*	Carbohydrates, proteins	8	4	0.005	NR	50–200
Parniakov et al., 2015	*Nannochloropsis* sp.	Phenols, chlorophylls	20	20	0.01	400	NR
t‘Lam et al., 2017	*C. vulgaris, N. oleoabundans*	Proteins	1.6–3	2	0.05–5	1–40	120–964
Safi et al., 2017	*N. gaditana*	Proteins	1.6–13.5	NR	5	2 or 10	NR
Carullo et al., 2018	*C. vulgaris*	Carbohydrates, proteins	20	4	1–10	NR	1–1000
Bodénès et al., 2019	*C. reinhardtii*	Lipids	0–0.7	1	0.005–0.5	10	10

*NR, not reported; C. reinhardtii, Chlamydomonas reinhardtii; C. vulgaris, Chlorella vulgaris; H. pluvialis, Haematococcus pluvialis; N. gaditana, Nannochloropsis gaditana; N. oleoabundans, Neochloris oleoabundans; N. salina, Nannochloropsis salina.*

In this paper, we investigate, evaluate and compare ultrasound, freeze-thawing, and electroporation as three non*-*thermal, solvent-free approaches for extraction of proteins from the microalga *Chlorella vulgaris*; we use chemical lysis as the positive control (maximal achievable extraction), and absence of extraction treatment as the negative control. We demonstrate that electroporation allows largely debris-free extraction of proteins and lipids from microalgae, with feasible growth regeneration.

## Materials and Methods

### Algal Growth in Photobioreactor

Unicellular microalga *Chlorella vulgaris* SAG 211-11b was inoculated in 50 ml flasks. When cell culture reached stationary phase, cells were transferred to a 20 L laboratory photobioreactor for 2 to 3 weeks (the tubular part of bioreactor at illumination 300–350 μmol photons/m^2^s, flow rate 720 mL/min). The alga was grown in Jaworski’s medium (Schlösser, 1982) with 16 h illumination per day, constant mixing, and automated measurements of temperature (kept in the interval 22 ± 2°C), O_2_ concentration (kept above 7 mg/L) and pH value (kept in the interval 6.5–7.5) every 10 min. Every second day, cell density, morphology and potential cell malformalities were checked under the microscope. Nutrient concentrations (NO_3_^–^, PO_4_^3–^, SO_4_^2–^) were also monitored spectrophotometrically (HI83225, Hanna Instruments). To avoid limitation of protein synthesis due to limited availability of nitrogen in the media, nitrogen source (NaNO_3_ at concentration 124 mg/L) was added when nitrogen values dropped below 70% of initial concentrations (usually every 7–10 days of cultivation).

### Extraction

**FIGURE 1:**
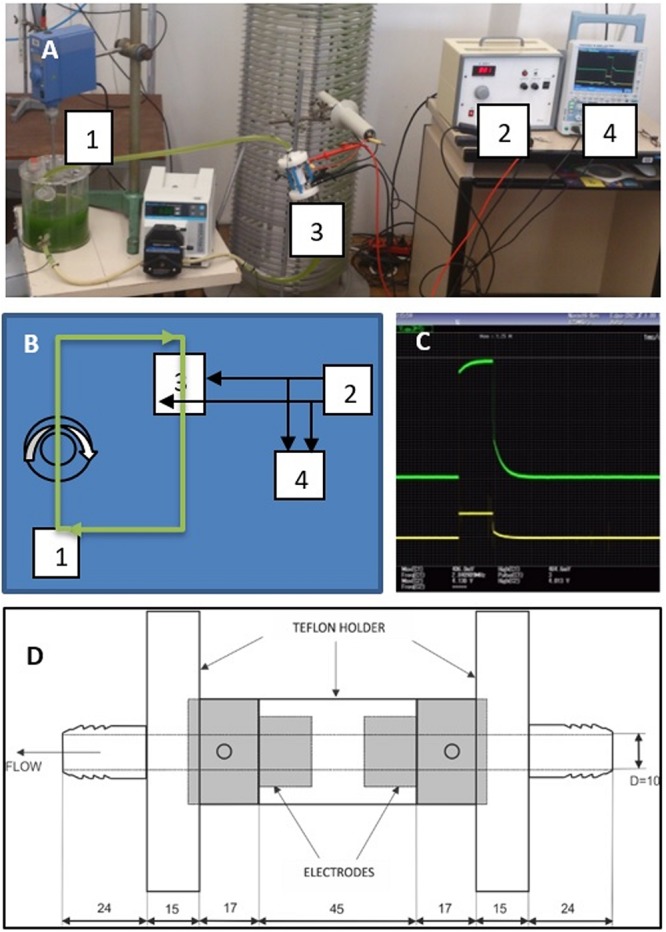
**(A)** The setup: algal photobioreactor (1) coupled with electroporation device (2) through the flow chamber (3), and electric pulses monitored by oscilloscope (4). **(B)** Schematic presentation of the components as labeled in panel **(A)**. **(C)** An psciloscope snapshot of the monitored unipolar square wave pulse for the setting EL4 as specified in Table 2 (green curve: voltage reaching the amplitude of 4 kV; yellow curve: electric current reaching the amplitude of 550 mA). **(D)** The flow chamber for electroporation of the algal suspension, with the gray areas representing the electrodes (design by Gianpiero Pataro, University of Salerno-UNISA).

When algal culture was reaching the end of exponential growth phase (in our conditions at app. 10^7^ cells/mL), aliquotes of algal suspension (8 × 0.5 L) were used for each of the treatments, with their quantitative parameters and abbreviations used henceforth specified in Table 2. For electroporation, the experimental setup, a snapshot of monitored unipolar square wave pulse voltage and current, and the flow chamber are schematically presented in Figure 1. The conductivity at the beginning of the experiments was in the range 185–273 μS/cm. All treatments were performed at room temperature, except ultrasonication where samples were put on ice during the exposure to avoid excessive heating.

**TABLE 2:** Extraction treatments used.

**Abbreviation**	**Treatment**	**Exposure and parameters for 0.5 L of algal suspensions (0.4 g d.w./L of suspension)**	
NC	Negative control	Algal suspension left on the laboratory desk for 30 min	
PC	Positive control	Detergent added (2,5% sodium dodecyl sulfate, Sigma-Aldrich) for 10 min	
US	Ultrasonication	Ultrasonic homogenizer Cole and Parmer, Chicago Ilinois, 40 W for 10 min, on ice in parts of 50 ml at once	
FT	Freeze-thawing	3 times slow freezing overnight at −20°C and thawing at 22°C	
EL1	Electroporation 1	Circulating in electro flow chamber, flow 0.72 L/min, 10 Hz, 30 min.	3 kV, 100 μs, i ≈ 300 mA
EL2	Electroporation 2		4 kV, 100 μs, i ≈ 550 mA
EL3	Electroporation 3		3 kV, 1 ms, i ≈ 400 mA
EL4	Electroporation 4		4 kV, 1 ms, i ≈ 550 mA

After every treatment, algal suspension was centrifuged (5000 rpm, 10 min) to allow the algal pellet (i.e., the centrifugate) and the supernatant to be analyzed separately. Cell density, conductivity, temperature, dry weight and protein and lipid content were measured before and after each treatment (after treatment separately for supernatant and algal pellet). Cell density and morphology were assessed microscopically with Bürker-Türk hemocytometer. Conductivity and temperature were measured with a multi sonde (Multi 3420 SETF, WTW). Dry weight was measured by weighing glass fiber filters (Sartorius GF/C) before and after filtration of 100 ml of algal suspensions with a known number of cells after drying at 105°C for 2 h. Dry weight was compared to weight of liophylised algal culture to validate the dry weight data.

### Extracted Proteins Quantification

Extracted protein content was quantified by a modified Bradford Assay (Bradford, 1976). Protein samples with protein content in range 1–25 μg were diluted with distilled water to final volume of 900 and 200 μl of Bradford reagent was added and mixed. The absorbance at 595 nm against blank sample (900 μl of distilled water + 200 μl Bradford reagent) was measured after 5 min incubation. The protein amount was determined from the calibration line prepared using solutions of bovine serum albumin (BSA) in amounts ranging from 1 to 25 μg.

### Extracted Lipids Quantification

Lipids released from algae into the non-polar phase were quantified by measuring liquid component concentrations. Each sample of supernatant was vigorously shaken, and a volume of 50 mL was pipetted into a 250 mL flask, to which 50 mL of a mix (1.6:1 volume ratio) of n-hexane (LiChrosolv, Merck) and isopropanol (LiChrosolv, Merck) was added. Immediately after preparation, all samples of algal pellets were shaken intensely in isopropanol for at least 5 min, and then treated in an ultrasound bath unit for 1 h, where the temperature of samples reached 35°C. Over the night, they were left at room temperature. In the morning, the samples were intensely shaken again and treated in ultrasound bath for another hour. After 5 h of samples rest at room temperature, 3–4 separated phases were visible. In all cases 1 mL of the present most upper phase was filtered through 2 μm syringe filter (Chromafil O-20/25 PTFE, Macherey-Nagel) into the 1.5 mL HPLC vial. The presences of lipid molecules were confirmed by HPLC as described elsewhere (Likozar and Levec, 2014a, b; Likozar et al., 2016).

## Results and Discussion

### Cultivation in Photobioreactor for High Biomass Yield

Our cultivation method in photobioreactor for green alga *Chlorella vulgaris* led to high biomass yield (up to 0.4 g/L) which is the basic prerequisite for an extraction technique to be deemed efficient. Balanced nutrient composition and non-limiting source of nitrogen are key elements for maximal protein production, therefore it was assumed reasonable to add nitrogen source during algal growth when nitrogen values in surrounding media dropped under 70% of initial concentrations. For all experiments, direct algal suspension was used, with no concentration of cells prior to extraction. Elemental composition of the medium used and of the main nutrients in *C. vulgaris* are shown in Table 3.

**TABLE 3:** The elemental composition medium composition and main nutrients (C, carbon; O, oxygen; H, hydrogen; N, nitrogen; P, phosphorous; Na, sodium; Mg, magnesium; S, sulfur) in *C. vulgaris*.

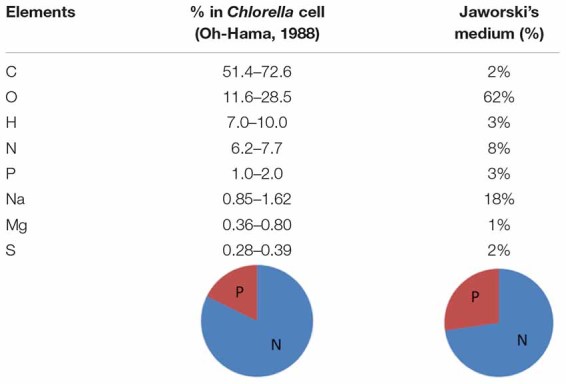

### Protein Extraction

**FIGURE 2:**
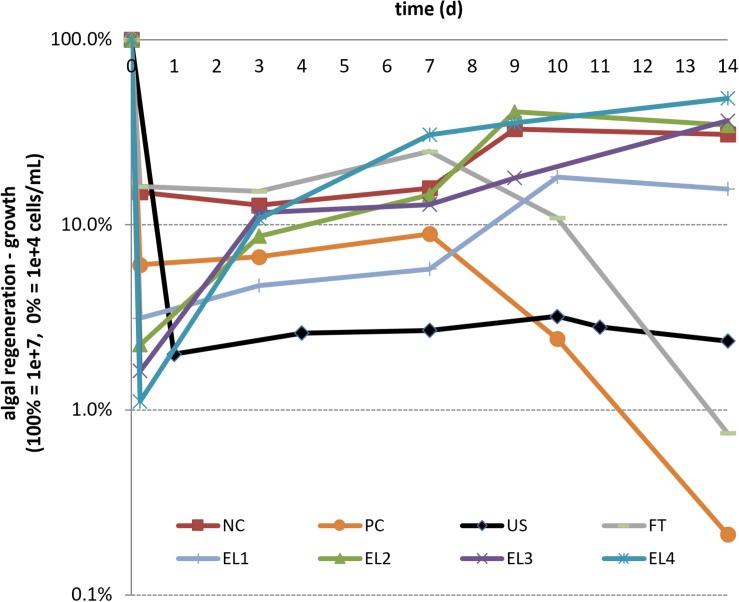
Growth regeneration (average values, calculated from cells per ml ranging from max 1e + 07 = 100% to min 1e + 04 = 0%) after different extraction techniques (abbreviations as in Table 2). The first assessment was performed at about 0.5 h after the treatment. After the lag growth phase of several days, the populations of electroporated algal cells (EL1–EL4) resumed growth with the same dynamic as control cells, while no such effect was observed with PC, FT, or US treatment.

The decrease and growth regeneration after the treatments is shown in Figure 2. Since microscopic observations can not detect all cell changes or injuries and each round green shape is counted as a cell, for further experiments flow cytometry as cell counting method should be employed, providing additional information about cell size, granulation and autofluorescence of photosynthetic pigments. Temperature and conductivity measurements are presented in Table 4.

**TABLE 4:** Average temperature (°C) and conductivity (μS/cm) ± standard deviation, before and after every treatment.

Abbreviation *	**Treatment**	**Conductivity**		**Temperature**	
		**Before**	**After**	**Before**	**After**
NC	Negative control	223.0 ± 36.6	225.5 ± 34.1	22.4 ± 0.5	22.4 ± 0.5
PC	Positive control	225.7 ± 44.4	1715.7 ± 862.4	22.3 ± 0.3	23.7 ± 0.6
US	Ultrasonication	222.0 ± 4.2	224.8 ± 5.9	21.7 ± 0.9	26.6 ± 3.4
FT	Freeze-thawing	222.0 ± 4.2	232.7 ± 10.3	21.6 ± 0.6	22.0 ± 0.0
EL1	Electroporation 1	227.5 ± 36.4	237.3 ± 33.0	23.1 ± 0.9	25.1 ± 0.7
EL2	Electroporation 2	228.0 ± 35.3	242.5 ± 30.8	22.6 ± 1.4	25.2 ± 0.2
EL3	Electroporation 3	227.0 ± 45.3	263.0 ± 41.1	22.3 ± 1.5	29.6 ± 2.0
EL4	Electroporation 4	226.7 ± 44.0	278.3 ± 53.4	22.3 ± 1.5	35.0 ± 5.1

*see Table 2 for treatment parameters.

**FIGURE 3:**
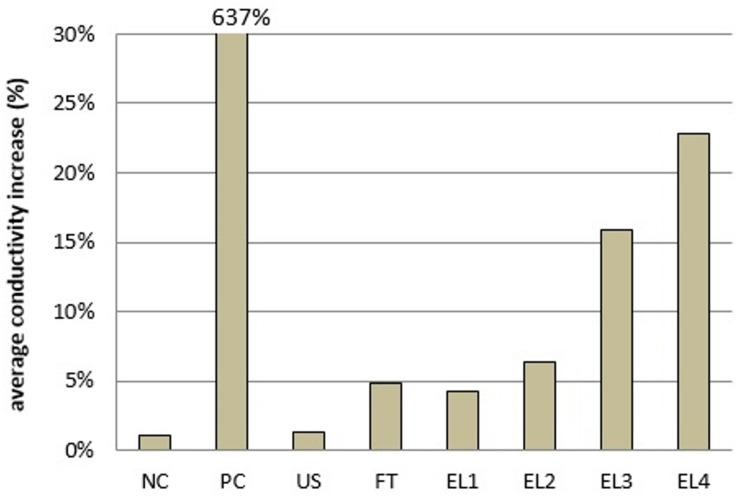
Average electrical conductivity increase for different extraction treatments. NC, negative control; PC, positive control; US, ultrasound; FT, freeze-thaw; EL, electroporation (abbreviations EL1–EL4 as in Table 2).

After each of the investigated extraction treatments, the conductivity increased; in the positive control (PC) with detergent addition, conductivity increased more than 6-fold (by 637%), while after the electroporation, conductivity increased proportionally to the energy input; except for PC, the increase was the highest with EL4 at 23% (Figure 3). Membrane conductivity and cell suspension conductivity increased (Table 4), due to ions release from cells into the medium due to concentration gradient and membrane increased permeability, which is a consequence of electroporation (Pavlin et al., 2005).

Similarly to conductivity, after electroporation also temperature increased proportionally to the energy input; the temperature increase was the highest with EL4 at 13 ± 6°C (Table 4). After centrifugation there was no difference in algal pellets (centrifugates) between different treatments; the pellets represented on average 4 ± 1% of total volume (and 82 ± 15% of total weight) of algal culture, which has been treated with US, FT, EL1, EL2, EL3 or EL4.

Protein content (in mg/L of algal suspension) was calculated to the measured dry weight of algal cultures for every treatment, separately for pellet and supernatant (Table 5). In all treatments except US, the mass of the pellet and supernatant were of the same order of magnitude, while with US the pellet was much smaller, reflecting much higher degree of cell fragmentation into debris too small to settle into a pellet upon centrifugation, and resulting in the much higher relative protein content in the supernatant relative to the pellet. In EL1–EL4, Western blot with Coomassie staining performed on the supernatant revealed that the extracted proteins sizes were between 35 and 55 kDa.

**TABLE 5:** Protein (P) content in mg/L of algal suspension and g/g dry weight of algal cultures, separately for pellet (p) and supernatant (s) of different treatments.

	**Average P**		**Average P**		
	**(mg/L)**		**(g/g dry weight)**		**Relative P (%)**
Treatment*	**p**	**s**	**p**	**s**	**s:p**
NC	150.0	7.9	2.9	0.3	11
PC	203.9	89.7	3.9	1.7	43
US	166.5	13.4	2.1	4.9	237
FT	68.1	3.3	2.0	0.2	12
EL1	211.7	11.4	4.0	0.5	12
EL2	168.3	11.8	2.4	0.6	25
EL3	136.9	12.5	2.2	0.7	32
EL4	149.5	13.1	2.3	0.9	38

*see Table 2.

**FIGURE 4:**
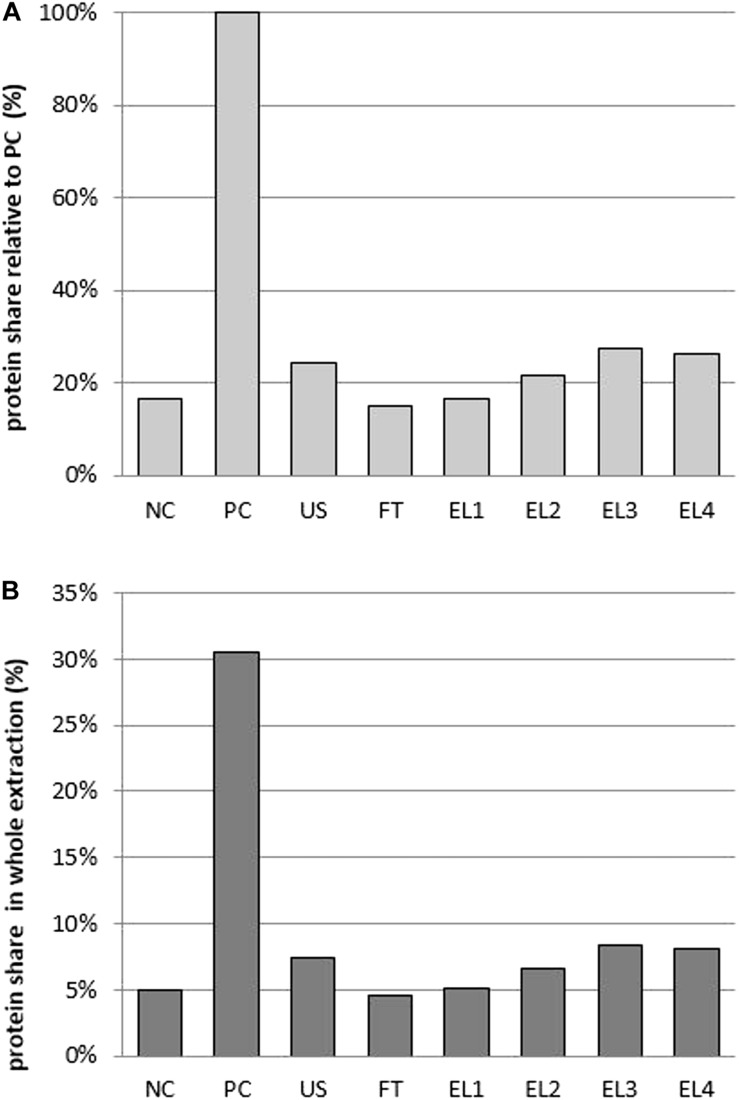
**(A)** The fraction of extracted protein concentration relative to the positive control. **(B)** The fraction of the total proteins extracted into the supernatant. NC, negative control; PC, positive control; US, ultrasound; FT, freeze-thaw; EL, electroporation (abbreviations EL1–EL4 as in Table 2).

The highest concentration of extracted proteins was obtained in the positive control (PC – chemical lysis), with EL2-EL4 reaching up to 27%, US up to 24%, and FT up to 15% of the yield obtained in PC, respectively (Figure 4A). The fraction of the total proteins extracted into the supernatant was also the highest in PC at 30% (with the remaining 70% in the pellet), while with EL2–EL4 it was up to 8%, US up to 7%, and FT up to 5% of the total proteins extracted (Figure 4B). While these yields may seem poor, we must bear in mind that PC – especially with alkaline solvents – dissolves not only the cell membrane but also the cell wall, resulting both in release of previously bound proteins into the supernatant and in complete disintegration of the cell that litters the extract with cells’ structural debris. The latter is largely the case also for US, and partly for FT, while in EL the permeabilizing effect can be limited and reversible, with the cells retaining their viability and integrity, and the extract is largely free of this debris.

### Algal Growth Regeneration

Successful treatment for extraction of substances from algal cells, where a subpopulation of cells would still remain viable, is based on compromise to disrupt the algal cells sufficiently as to extract compounds, yet mildly enough as to not kill all the cells (reversible permeabilization of algal membrane). In this way, the source of biomass, water and nutrients is recyclable, making the approach non-destructive and contributing to the sustainability of resources for a growing world population (Buchmann et al., 2019). Our findings indicate that after the lag growth phase of a few days, the population of electroporated algal cells grows with the same dynamics as control cells, while this in not true for PC, FT, and US treatment, where growth regeneration could not be reached (see Figure 2). Certainly, the viable fraction of biomass also contained reversibly electroporated microalgae, from which there was also some extraction/leakage into the supernatant, which contributed to the initial lag and gradual approach of post-exposure growth rate to the pre-exposure levels (and thus to the biomass regaining its full quality and functionality).

### Simultaneous Protein and Lipid Extraction

In some end-user applications of value-added algal substances, it is desired that proteins and lipids are extracted at the same time (e.g., in mixed algae extracts used as a renewable agriculture or aquaculture feedstock). Thus for electroporation as the most yield-efficient and the only non-destructive among the considered solvent-free protein extraction techniques, two preliminary experiments of simultaneous protein/lipid extraction were also investigated, by HPLC, using the same electroporation protocols as above. Although in comparison to glyceryl trioleate model, as a HPLC standard, up to six different oleaginous peaks could be detected in the supernatants of electroporated algal cultures (none in other control samples), the quantities were relatively low, with the fraction of extracted lipids in the supernatant comprising only up to 7% of their total extracted mass (with the remaining 93% in the pellet). As anticipated, positive control revealed 21 different peaks, since cells were lysed with detergent (Table 6). Interestingly, the cumulative lipid quantities in NC pellet sample were up to 6.6 g/L, but for electroporation, up to 27.5 g/L (Table 6). Increasing the time after electroporation treatment process, combined with centrifugation, may increase yields further. Regarding components, tri-, di- and mono-glycerides were obtained, as well as other fatty acid derivatives, with the distribution of the latter affected by culture. Fractionation of proteins and lipids from the extract could be performed by separation of the water-soluble fraction containing proteins, and subsequent extraction of lipids from the residual by elution in ethanol (Golberg et al., 2016).

**TABLE 6:** Concentration (g/L) and number of different lipid substances (HPLC peaks) extracted from *C. vulgaris* after electroporation, separately for pellet and supernatant; NC negative control, PC positive control.

Treatment*	**Number of peaks in pellet**	**Lipids (g/L) in pellet**	**Number of peaks in supernatants**	**Lipids (g/L) in supernatants**
NC	13	6.6	0	0
PC	21	39.1	0	0
EL1	9	21.6	6	1.2
EL2	8	18.1	5	0.7
EL3	13	27.5	5	0.7
EL4	4	9.6	5	0.7

*see Table 2.

## Conclusion

Electroporation, though lower in extraction yields than chemical lysis or mechanical disintegration, is in contrast to them a technique for largely debris-free extraction of proteins from microalgae, with no need for prior concentration or drying, with feasible growth regeneration, and with potential for simultaneous extraction of intracellular algal lipids into the supernatant. While we demonstrated the use of electroporation for extraction of proteins and lipids as primary microalgae products, for the scope of biorefineries other studies have shown it is a promising method for simultaneous extraction of various additional valuable products, ranging from vitamins and carbohydrates (Mahnič-Kalamiza et al., 2014; Postma et al., 2016) to phenols and chlorophylls (Parniakov et al., 2015). This approach furthermore has large potential for upscaling, which is important for industrial use (Kotnik et al., 2015). Regarding the extent of regeneration, further lifecycle analysis will be necessary for it to be broadly recognized. As irreversible electroporation also inactivates microorganisms including pathogens, this approach can simultaneously reduce the risk of infection that is otherwise a common biotechnological problem (Rego et al., 2015).

## Data Availability Statement

All datasets generated for this study are included in the article/supplementary material.

## Author Contributions

TE, JG, and DM conceptualized the study. KF developed the generator of electric pulses. TE and MK conducted the experiments, analyzed the data and interpreted the results on protein extraction. BL conducted the experiments, analyzed the data and interpreted the results on lipid extraction. DM supervised the experimental work. DM and TK critically revised the results, their interpretation, and improved the manuscript with important intellectual content. All authors have read and approved the submitted manuscript.

## Conflict of Interest

The authors declare that the research was conducted in the absence of any commercial or financial relationships that could be construed as a potential conflict of interest.
